# The epidemiology of rotavirus disease in under-five-year-old children hospitalized with acute diarrhea in central Uganda, 2012-2013

**DOI:** 10.1007/s00705-015-2742-2

**Published:** 2016-01-02

**Authors:** Josephine Bwogi, Samuel Malamba, Brian Kigozi, Prossy Namuwulya, Phionah Tushabe, Sarah Kiguli, Denis Karuhize Byarugaba, Ulrich Desselberger, Miren Iturriza-Gomara, Charles Karamagi

**Affiliations:** Uganda Virus Research Institute, 51-59 Nakiwogo Road, P.O. BOX 49, Entebbe, Uganda; Department of Paediatrics and Child Health, College of Health Sciences, Makerere University, P.O. BOX 7072, Kampala, Uganda; Department of Microbiology, College of Veterinary Medicine and Biosecurity, Makerere University, P.O. BOX 7072, Kampala, Uganda; Department of Medicine, University of Cambridge, Cambridge, CB2 0QQ UK; Department of Clinical Infection, Microbiology and Immunology, Institute of Infection and Global Health, University of Liverpool, Ronald Ross Building, West Derby Street, Liverpool, L69 7BE UK

**Keywords:** Rotavirus, Genotyping, Mixed infections, Reassortment, Food hygiene, Zoonotic transmission

## Abstract

A cross-sectional study was undertaken during 2012-2013 to determine the prevalence, strains and factors associated with rotavirus infection among under-5-year-old children hospitalized with acute diarrhea in Uganda. Rotaviruses were detected in 37 % (263/712) of the children. The most prevalent strains were G9P[8] (27 %, 55/204) and G12P[4] (18.6 %, 38/204). Mixed infections were detected in 22.5 % (46/204) of the children. The study suggests that consumption of raw vegetables (OR = 1.45, 95 % CI = 1.03-2.03) and family ownership of dogs (OR = 1.9, 95 % CI = 1.04-3.75) increases the risk of rotavirus infection. The study findings will be used to assess the impact of RV vaccination in Uganda.

Diarrhea is a major cause of mortality in children under 5 years of age in developing countries, contributing up to 21 % of deaths [1]. Rotavirus (RV) is the main cause of diarrhea in children worldwide [2, 3]. Globally, RV causes more than 450,000 deaths annually in children below five years, with 80 % of deaths occurring in sub-Saharan Africa and South Asia [4].

RVs are classified into eight major groups (A-H) [5]. Group A RVs cause most infections seen in young children. Globally, G1, G2, G3, G4, G9 and G12 in combination with P[8] or P[4] constitute 88 % of the circulating strains [6]. In Africa, other G/P type combinations such as G8P[6], G8P[8] and G12P[6] are prevalent [7]. The great diversity of RV strains in developing countries is believed to originate from interspecies transmission in societies where contact with animals is frequent [8].

RV diarrhea causes severe dehydration, leading to high mortality in developing countries [4]. It is common in children attending day care, and living in households or neighborhoods with children suffering from diarrhea is a known risk factor [9–11], suggesting that RV is mainly spread via person-to-person transmission.

In countries where RV vaccination has been introduced, a decrease in RV-associated morbidity and mortality has been observed [12]. However, the efficacy of the two currently licensed live attenuated vaccines is lower in developing than in developed countries [13]. Whereas this reduced vaccine efficacy has been attributed to the wide diversity of circulating RV strains in developing countries, genomic studies have not consistently supported this hypothesis [14].

In Uganda, diarrhea is among the top four causes of morbidity in infants and young children [15], and RV is responsible for 33 % to 45 % of diarrhea cases [16]. In anticipation of the introduction of routine RV vaccination in Uganda, this study was undertaken to collect baseline data on RV infections in children hospitalized with acute diarrhea and on RV transmission in households. The study findings will be used to assess the impact of RV vaccination on rotavirus-associated disease burden and RV strain distribution in Uganda.

From September 2012 to September 2013, a cross-sectional study was carried out in which stool samples and venous blood were collected from 712 children (2-59 months) hospitalized with acute diarrhea in four hospitals in central Uganda. Acute diarrhea was defined as three or more watery stools in 24 hours and having started less than 14 days before admission. Children with bloody diarrhea were excluded from the study. Stool samples were also taken from 254 household contacts who were either mothers or caregivers (65.7 %, 167/254) and female (78.7 %, 200/254). Among the household contacts, 10/254 (3.9 %) had diarrhea in the three weeks prior to recruitment. Stool samples were also collected from 40 domestic animals (18 goats, 11 pigs, 7 dogs and 4 cattle) with and without diarrhea symptoms, although only one animal (2.5 %) had diarrhea in the two weeks prior to recruitment. The household contacts and animals were recruited from the homes of the children that tested RV positive. A structured questionnaire was used to collect demographic, clinical and socio-economic data of the patient, including feeding habits (whether breastfeeding or not, consuming milk of animal origin, eating raw vegetables or fruits in the week prior to admission, drinking water source), hand hygiene, attendance of daycare centers or kindergartens and contact with animals.

Stool samples from symptomatic children and from household animals were examined for RV using ELISA (ProSpecT, Oxoid Ltd, Hampshire, UK), following the manufacturer’s instructions, at the microbiology laboratories of Mulago and Masaka hospitals or the Uganda Virus Research Institute (UVRI). Molecular assay detection and typing assays were carried out at the Expanded Program for Immunization (EPI) Laboratory of UVRI.

RV RNA was extracted and transcribed to cDNA using previously described methods [17, 18]. A sensitive NSP3 specific real-time RT-PCR [19] was used to enable detection of low RV loads associated with asymptomatic shedding in household contacts. RV-positive stool samples were G- and P-genotyped using a nested RT-PCR as described previously [18, 20–22].

Malaria parasites were screened for on Giemsa-stained blood smears. Screening for HIV infection was done using an antibody test (Alere Determine™ HIV 1/2 test). Children below 18 months of age who tested positive on the Alere Determine™ HIV 1/2 test were also tested by HIV PCR test [23].

Data were analyzed using STATA 12.1 (StataCorp LP, USA). Logistic regression models were used to assess bi-variate associations between the dependent variable (RV status) and the independent variables. Factors with a *p*-value of ≤0.1 in the bivariate analysis and factors of biological plausibility were included in the multi-variable analysis, adjusting for age. All factors with a two-sided *p*-value of ≤0.05 were considered statistically significant and were included in the final model.

The study was approved by the Research and Ethics Committees of School of Medicine, College of Health Sciences, Makerere University; Uganda Virus Research Institute; Mulago National Referral Hospital; St. Francis Hospital Nsambya; and Uganda National Council of Science and Technology.

Demographic characteristics of the sample are given in Table 1. RV was detected in 263 (37 %) of the 712 hospitalized children. However, these results may represent the more-severe spectrum of the disease. The RV results were similar to what has been reported in other countries that have not yet introduced routine RV vaccination. RV diarrhea cases presented all year round with no clear seasonal variation. This was unlike what has been observed elsewhere [24, 25], probably due to the location of Uganda on the equator.

**Table 1:** Socio-demographic characteristics of under-five-year-old children hospitalised with acute diarrhea in central Uganda, 2012–2013

Characteristic	Total N = 712	Rotavirus positive N (%)	Chi-square *p*-value
**Age of child (months)**			
≤6	139	45 (32.4)	0.133
6.1-12	345	136 (39.4)	
12.1-24	189	73 (38.6)	
Above 24	39	9 (23.1)	
**Sex**			
Male	421	159 (37.8)	0.581
Female	291	104 (35.7)	
**Vesikari score**			
Moderate (7-10)	39	16 (41)	0.586
Severe (≥11)	673	247 (36.7)	
HIV infection**			
Negative	658	249 (37.8)	0.042
Positive	34	7 (20.6)	
Malaria infection**			
Negative	673	252 (37.4)	0.237
Positive	33	9 (27.3)	
Ate raw vegetables in past week**			
No	434	149 (34.3)	0.080
Yes	269	110 (40.9)	
Caretaker washes hands with soap after toilet**			
No	39	20 (51.3))	0.062
Yes	629	229 (36.4)	
**Treatment of patient’s drinking water**			
Yes	687	252 (36.7)	0.811***
No	12	4 (33.3)	
Animals drinking from patient’s water source**			
No	650	238 (36.6)	0.817
Yes	47	18 (38.3)	
**Patient’s family owns dog(s)**			
No	666	240 (36.0)	0.058
Yes	46	23 (50)	
**Patient’s family owns animals**			
Yes	122	52 (42.6)	0.153
No	590	211 (35.8)	

** The number of respondents for this variable was less than 712

*** Fisher’s exact *p*-value

Two hundred fifty-four (94.5 %) of the RV-positive children were ≤24 months old. Six hundred seventy-three children (94.5 %) had severe illness as determined by a Vesikari score ≥11, and among these, 36.7 % (247/673) were RV positive. There was no statistical difference (*p* = 0.586) between the Vesikari scores in RV-positive and RV-negative children. Most RV-positive cases were associated with hospitalization lasting ≤5 days (*p* = 0.001), but there were no deaths or referrals recorded among children admitted with RV diarrhea. HIV infection was detected in 4.9 % (34/692) of the children, of which 7/34 (20.6 %) had RV diarrhea. HIV-positive children were less likely to have RV diarrhea (*p* = 0.042). A previous study in Uganda found no association between HIV and RV diarrhea [16]. Malaria parasites were detected in 4.7 % (33/706) of the children, of which 9/33 (27.3 %) had RV diarrhea. Generally, the small number of children with RV diarrhea who had the risk factors may have affected the observations of the association between the risk factors and RV diarrhea (Table 1).

In the multi-variable analysis the factor that was associated with RV diarrhea was consumption of raw vegetables in the week prior to recruitment (OR = 1.45; 95 % CI: 1.03-2.03, *p* = 0.032). Although RV transmission is primarily from person to person, it is likely that fecal contamination of fresh produce may have played an important role in transmitting the virus [26]. This route of transmission is compatible with the greater strain diversity observed in low-income countries and also with the high proportions of co-infections with multiple RV strains, which also facilitate the emergence of reassortant strains. Analysis of foods consumed raw for the presence of RV and other markers of fecal contamination would be required in order to fully evaluate the role of foodborne transmission on rotavirus-associated diarrhea.

Although children from families owning dogs were apparently twice as likely as those not owning dogs to have RV diarrhea (OR = 1.98; 95 % CI: 1.04-3.75, *p* = 0.037), none of the examined animal stool samples were positive for RV. Molecular studies are thus needed to determine the role of zoonotic transmission of RV in this setting.

RV diarrhea was also less likely to be found in children whose age was >24 months (OR = 0.48; 95 % CI: 0.19 - 0.99, *p* = 0.049) and those who were hospitalized for more than 5 days (OR = 0.46; 95 % CI: 0.28 - 0.75, *p* = 0.002).

Out of the 263 RV-positive human samples, 204 (77.6 %) yielded genotyping results. The most prevalent RV genotypes in the hospitalized children were G9P[8] (27.0 %, 55/204) and G12P[4] (18.6 %, 38/204) (Table 2). Although G9P[8] has continued to be among the most prevalent genotype circulating in Uganda over the years, G12P[4] strains, which had not previously been reported in Uganda [7, 27], were found at a high frequency. G12 RVs, which were first detected in 1987, have recently spread worldwide [28]. Although G12 RVs have been detected primarily in association with P[6] and P[8] types in humans, their origin remains unclear. G12 RVs have been reported sporadically in pigs, but sequence analysis has shown that human and porcine G12 strains belong to different genetic clusters [29]. Mixtures of RV genotypes were highly prevalent. A total of 46/204 (22.5 %) samples contained mixtures of different RV strains. They contained mostly the same G- or P-types identified in single-strain infections. They had one G-type with two or more P-types (17.4 %, 8/46), two G-types with one P-type (71.7 %, 33/46), or two or more G- and P-types (10.9 %, 5/46). A total of 22/204 (10.8 %) samples were partially typed, and 2/204 (1 %) failed to be genotyped (Table 2). Unusual genotypes that may suggest an animal origin, such as G10 or P[10], were only detected among mixed infections. Although such mixed infections could provide conditions for the emergence of animal-human reassortant strains, no reassortants containing either of these two genotypes were detected.

**Table 2:** Rotavirus genotypes of children hospitalised with acute diarrhea in central Uganda, September 2012–September 2013

G-genotype													
P-genotype	G9	G12	G3,9	G3	G9,12	G3,12	G4,12	G1	G9,10	G3,9,12	Identified G-type only	No G-type identified	Total (%)
P[8]	55	11	4		3	1	2	1					77 (37.7)
P[4]	4	38	2	1	2								47 (23)
P[6]	8	3	17	13	1	1							43 (21)
P[4, 8]		3	1		1				1				6 (3)
P[8, 10]	2												2 (1)
P[4, 8, 10]	2												2 (1)
P[4, 6]						1							1 (0.5)
P[6, 8]	1												1 (0.5)
P[4, 6, 8]										1			1 (0.5)
Identified P-type only												4	4 (2)
No P-type identified											18	2	20 (9.8)
Total (%)	72 (35.2)	55 (26.9)	24 (11.8)	14 (6.9)	7 (3.4)	3 (1.5)	2 (1)	1 (0.5)	1 (0.5)	1 (0.5)	18 (8.8)	6 (3)	204 (100)

All household contacts and animal stool samples tested negative for RV. However, the number of animals tested was small, and a study with more animals is required to further study the association of RV diarrhea in children with family ownership of dogs.

Nevertheless, this study showed that RV infection is common in under-five-year-old children hospitalized with acute diarrhea in central Uganda, and the most frequently identified RV strains were G9P[8] and G12P[4].

Routine RV vaccination and improved food hygiene are urgently needed. After the introduction of RV vaccination, continued surveillance to monitor its impact on the prevalence and molecular epidemiology of RV strains as well as on foodborne transmission is recommended.
